# Acute pancreatitis with pancreatic gas gangrene

**DOI:** 10.1016/j.idcr.2023.e01859

**Published:** 2023-07-25

**Authors:** Kento Furuya, Naoya Itoh

**Affiliations:** a Department of Clinical Laboratory Medicine, Shizuoka General Hospital, Japan; b Division of Infectious Diseases, Aichi Cancer Center Hospital, Japan

**Keywords:** Pancreatic gas gangrene, Acute pancreatitis, *Clostridium perfringens*

## Case presentation

**Fig. 1:**
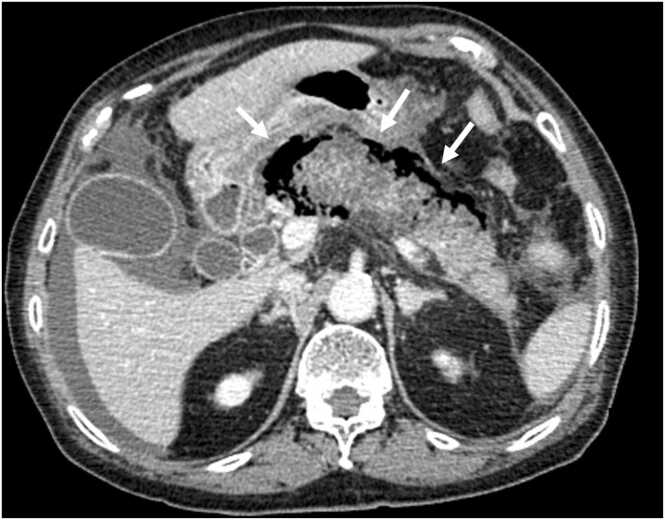
Contrast-enhanced computed tomography scan of an 83-year-old woman showing an enlarged pancreas and peripancreatic gas.

**Fig. 2:**
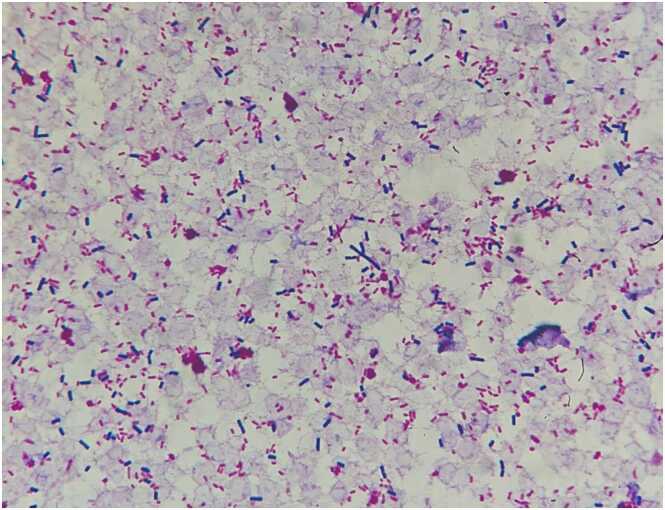
Smear of blood cultures demonstrating Gram-positive rods and Gram-negative rods.

An 83-year-old woman presented to our hospital with upper abdominal pain that had started the day before. Although her vital signs were normal during the visit, she developed shock one hour later. Physical examination revealed tenderness in her upper abdomen. Laboratory tests showed a white blood cell count of 23,100/µL, direct bilirubin of 2.7 U/L, amylase of 3739 U/L, pancreatic amylase of 3723 U/L, and lipase of 4796 U/L. Her abdominal contrast computed tomography showed an enlarged pancreas with surrounding gas (Fig. 1) and common bile duct stones, but no gastrointestinal perforation or ischemia was observed. After taking blood cultures, we started meropenem, vancomycin, and micafungin. Unfortunately, the patient died eight hours after her visit. Later, blood cultures revealed the presence of *Clostridium perfringens* and *Escherichia coli* (Fig. 2).

*C. perfringens* can cause necrotizing soft tissue infections as well as gas gangrene of the liver and spleen [1]. The present case describes a rare instance of pancreatic gas gangrene and acute pancreatitis caused by *C. perfringens* and *E. coli*. The pathogenesis of the disease has not yet been fully elucidated. *C. perfringens* and *Klebsiella pneumoniae* have been reported as causative agents of pancreatic gas gangrene [2], [3]. Although antibiotics are not typically indicated for acute pancreatitis, we should promptly administer antibiotics since peripancreatic gas suggests a severe infection. Moreover, treatment of pancreatic gas gangrene with antibiotics alone is insufficient [3]. Early surgical intervention is necessary in addition to antibiotics.

## Ethical approval

The parent’s consent was required for publication.

## Consent

Written informed consent was obtained from the patients for publication of this article.

## Funding

None.

## CRediT authorship contribution statement

**Kento Furuya:** Data curation, Investigation, Resources, Visualization, Writing – original draft. **Naoya Itoh:** Writing – review & editing.

## Conflicts of interest

The authors have no conflicts of interest to declare.

## References

[1] Meyer J., Dupuis A., Huttner B.D., Tihy M., Bühler L. (2019). Gangrenous gas necrosis of the spleen: a case report. BMC Infect Dis.

[2] Ishii Y., Tsuchiya A., Hayashi K., Terai S. (2020). Pancreas gas gangrene caused by Klebsiella pneumoniae. Intern Med.

[3] Sánchez-Gollarte A., Jiménez-Álvarez L., Pérez-González M., Vera-Mansilla C., Blázquez-Martín A., Díez-Alonso M. (2021). Clostridium perfringens necrotizing pancreatitis: an unusual pathogen in pancreatic necrosis infection. Access Microbiol.

